# Laser‐assisted hatching on clinical and neonatal outcomes in patients undergoing single vitrified Blastocyst transfer: A propensity score–matched study

**DOI:** 10.1002/rmb2.12366

**Published:** 2021-01-27

**Authors:** Yuji Endo, Shingo Mitsuhata, Momoko Hayashi, Yoshitaka Fujii, Hiroaki Motoyama

**Affiliations:** ^1^ IVF Center Kurashiki Medical Clinic Kurashiki Japan

**Keywords:** laser‐assisted hatching, live birth rate, neonatal outcomes, single vitrified blastocyst transfer

## Abstract

**Purpose:**

This study determined the effect of laser‐assisted hatching on the clinical and neonatal outcomes of single vitrified blastocyst transfer.

**Methods:**

From June 2014 to March 2018, 289 matched pairs after propensity score matching were analyzed. During the blastocyst warming procedure, a small section of the zona pellucida area in the empty perivitelline space was sliced off using multiple laser beams. The clinical and neonatal outcomes of the laser‐treated group and non‐treatment control were analyzed.

**Results:**

In the laser‐assisted hatching group, significantly higher rates of clinical pregnancy (40.8% vs 29.4%, *P* < .01) and live delivery (34.3% vs 22.5%, *P* < .01) were observed compared to the control group. Other variables such as the average gestational weeks, the sex of the baby, birthweight, or congenital malformations were found to have no significant differences in neonatal outcomes. Moreover, all babies were singleton live births.

**Conclusions:**

Single vitrified blastocyst transfer treated with laser‐assisted hatching increases the live birth rate and has no adverse effects on neonatal outcomes.

## Graphical abstract

Blastocyst warming in a dilution solution resulted in a large perivitelline space, which allowed a small section of the zona pellucida to be sliced easily and safely using a noncontact laser beam. The blastocyst was completely escaped from the zona pellucida after overnight culture and improved the clinical pregnancy and live birth compared with the control group.

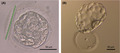

## 1 INTRODUCTION

Assisted hatching (AH) is a technique used to aid artificial blastocyst hatching from the zona pellucida (ZP). It is commonly used in artificial reproductive technology and is deemed to improve clinical results. Because hatching from the ZP is a critical process in implantation, AH was first introduced in clinical in vitro fertilization (IVF) in 1989.^1^ Over the years, researchers have studied the benefits of AH for couples experiencing infertility; however, this method needs to be examined further.^2^ According to The Practice Committee of the American Society for Reproductive Medicine (ASRM), AH procedures could improve the clinical pregnancy rate (CPR). However, they should not be used routinely for all IVF patients or patients with poor prognosis, due to the paucity of live birth data and the increased risk of multiple pregnancies.^3^ Many articles have described the efficiency of AH, but only a few studies have reported the live birth rate (LBR). The two previous meta‐analyses included 36 studies with 6459 patients and 31 studies with 5728 patients, respectively, but the researchers did not find a significant difference in the LBR between the AH treatment group and a control group.^4^, ^5^ A US multicenter analysis from the National ART Surveillance System focused on AH procedures from 35,518 cycles and found that AH was not associated with improved pregnancy outcomes.^6^ Also, the Society for Assisted Reproductive Technology Clinic Outcome Reporting System database found that AH slightly decreased the LBR in 151,533 first‐cycle, autologous frozen embryo transfers (FETs).^7^

However, data accuracy on this topic is still under examination. Many meta‐analyses did not evaluate the different techniques and protocols of AH, the different stages of the embryo, the type of cycle, or the indications for the procedure.^8^ This heterogeneity was not well discussed in previous publications; thus, there are conflicting views on the effectiveness of AH among researchers.

Alteri et al^8^ evaluated current AH protocols and found studies that support the benefit of ZP breaching in thawed blastocysts. The integration of a laser application system and frozen blastocyst transfer is an effective treatment that improves the IVF success rate, due primarily to the following advantages: (a) AH might overcome ZP hardening, which is caused by freezing‐thawing procedures and the prolonged embryo culture in vitro^9^, ^10^; (b) blastocyst transfer results in a more significant implantation potential than cleavage‐stage embryos owing to the improvement in embryo selection and embryo‐endometrium synchrony^11^, ^12^; and (c) frozen single blastocyst transfer results in a higher singleton LBR than a fresh single blastocyst transfer.^13^ The laser system has become widely used in AH because it is simple and less damaging to the embryos. However, the potential effects of using laser for frozen blastocyst have not been proven, and reports regarding the LBR are limited. Because the clinical findings on the LBR and neonatal outcomes are insufficient, we conducted a retrospective cohort study using propensity score analysis to investigate the references of single vitrified blastocyst transfer with laser treatment.

This study determined the effect of laser‐AH (LAH) on the clinical and neonatal outcomes of single vitrified blastocyst transfers among IVF patients.

## 2 MATERIALS AND METHODS

### 2.1 Experimental data and patient selection

**FIGURE 1:**
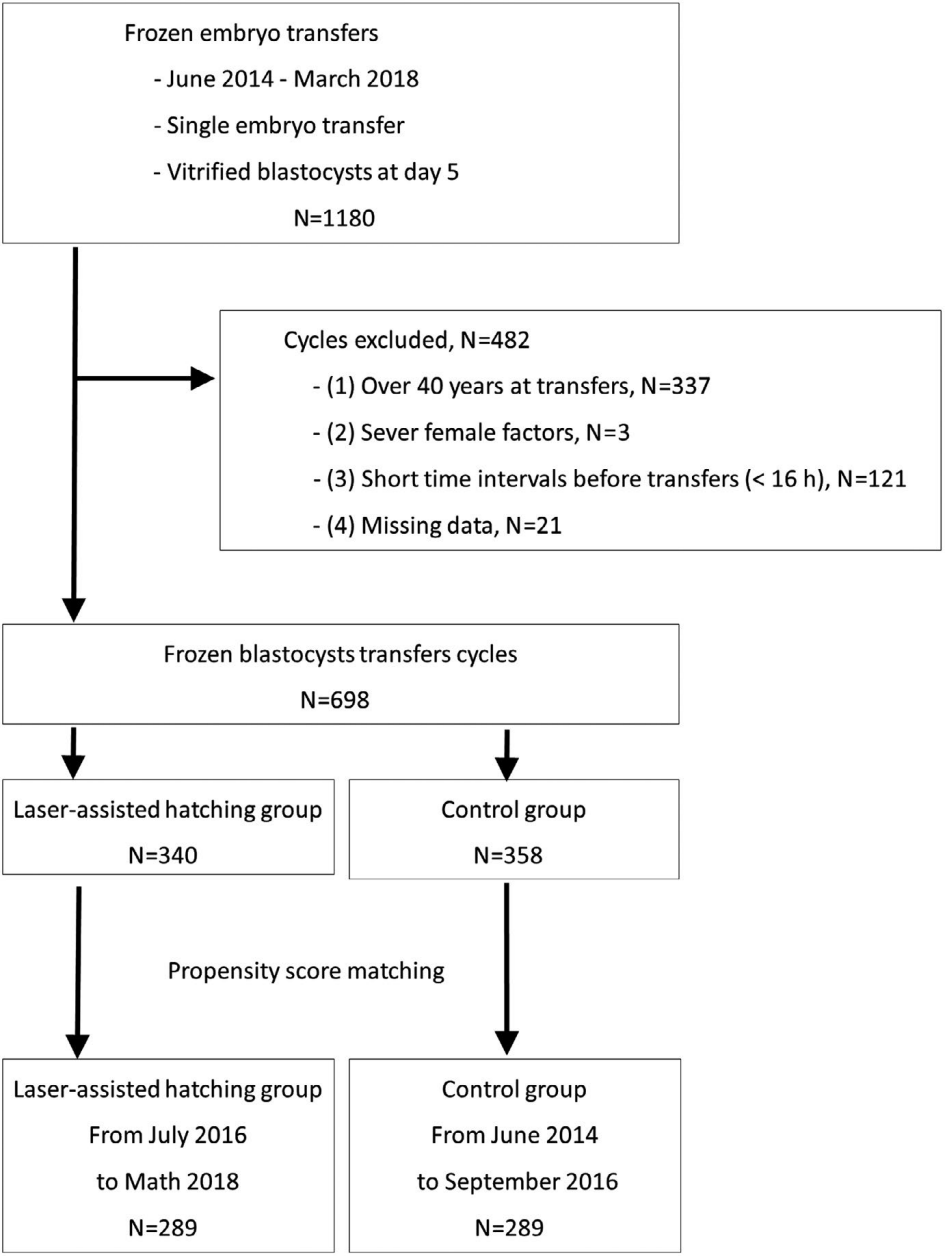
Patient inclusion flowchart

This study was a retrospective, observational study based on data collected from June 2014 to March 2018. Patients who had single vitrified blastocyst transfers were included if their day 5 blastocysts survived after warming, and they agreed to receive LAH. LAH was gradually performed on day 5 vitrified blastocysts in July 2016. The LAH group was from July 2016 to May 2018, and control from June 2014 to September 2016. The exclusion criteria were as follows: (a) patients older than 40 years, (b) patients with severe uterine factors or chromosomal abnormalities, (c) short time intervals before transfers (<16 hours), and (d) missing follow‐up data (Figure 1). The Kurashiki Medical Center and Ethics Committee approved this project, and informed consent was obtained in the form of opt‐out on the web site.

### 2.2 Embryo culture

In the oocyte retrieval cycle, ovarian stimulation was achieved using standard gonadotropin‐releasing hormone agonist/follicle‐stimulating hormone (FSH) protocols or an antagonist/FSH protocol. A vaginal ultrasound‐guided follicle puncture was conducted 36 hours after injection of human chorionic gonadotropin. The retrieved oocytes were inseminated by conventional IVF or intracytoplasmic sperm injection in accordance with a previously reported method.^14^, ^15^ The oocytes with two pronuclei and a second polar body at 17‐19 hours following insemination were defined as normally fertilized. They were cultured for 5 days in a medium (global^®^; Life Global) and supplemented with recombinant human albumin (G‐MM; Vitrolife) at 37°C in 6% CO_2_, 5% O_2_, and 89% N_2_.

### 2.3 Blastocyst vitrification and warming

All blastocysts were vitrified and warmed using Kitazato vitrification and warming media (Kitazato Company), as described previously.^16^ On day 5, some blastocysts were transferred in fresh cycles, and surplus viable blastocysts were vitrified using open‐system Cryotop^®^ (Kitazato Company). The blastocyst with a diameter greater than 150 µm was treated in 0.2 M hyperosmotic sucrose solution and mechanically punctured using a micro‐needle following a published report.^17^ The inner cell mass of the blastocyst was located at the 12 o'clock position, and the injection pipette punctured the blastocyst at the 3 o'clock position. The blastocoel fluid was completely aspirated with negative pressure, and then it was moved into the equilibration solution. The blastocyst was immersed in the equilibration solution within 15 minutes and sequentially exposed in the vitrification solution for 1.5 minutes. The blastocyst was immediately transferred to the Cryotop and submerged into the liquid nitrogen. After being stored in the storage tank for several months, the blastocyst was warmed in the thawing solution for 2 minutes and sequentially transferred to the dilution solution for 3 minutes. They were then transferred into the washing solution for 5 minutes. All were cultured overnight (≧16 hours), and the blastocyst that re‐expanded and having greater than 50% of the morphologically intact cells was considered as survival.

### 2.4 LAH procedure

**FIGURE 2:**
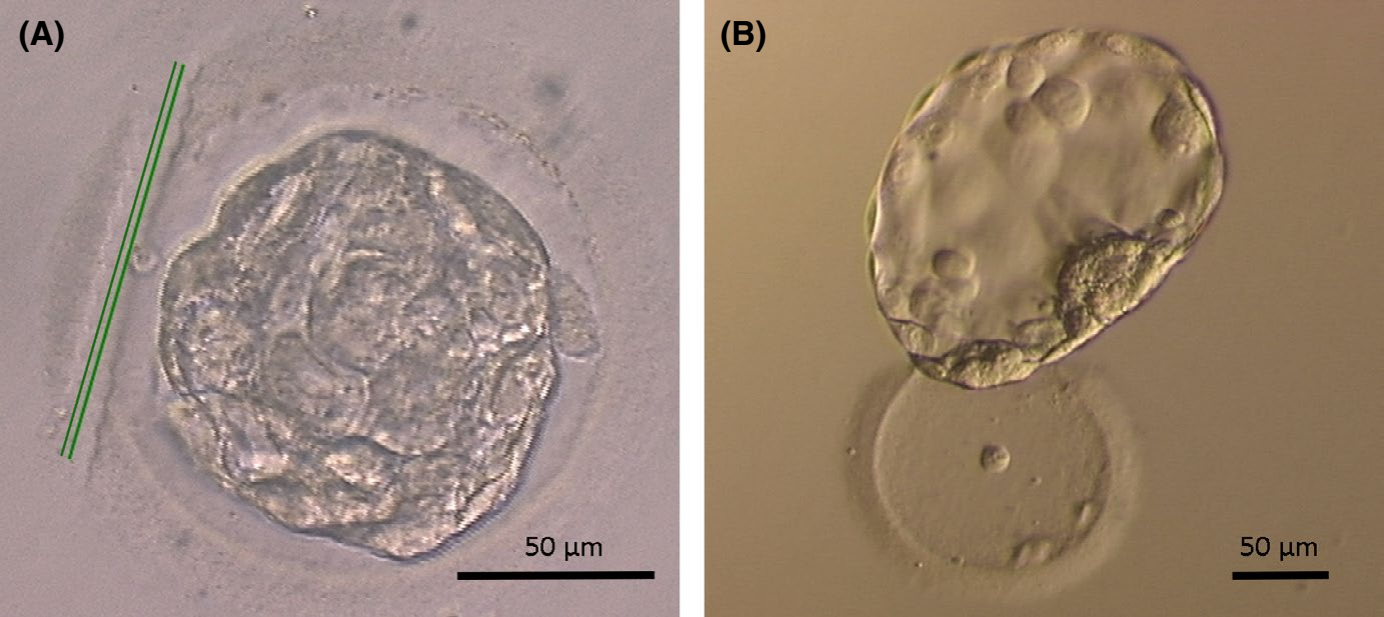
Laser‐assisted hatching procedure. A, AH was performed during the warming procedure in a dilution solution. A small section was sliced off the zona pellucida (ZP) area in the empty perivitelline space using 10‐15 laser beams (green double lines). B, Blastocyst completely hatched from ZP after overnight culture

Laser treatment (Saturn 5; Research Instruments Ltd.) was performed during the blastocyst warming procedure in the dilution solution. A small section of the ZP area, where the empty perivitelline space was found, was sliced off using 10‐15 laser beams (Figure 2). The laser pulse length was 999 µs, and a hole size was 18 µm.

### 2.5 Blastocyst scoring and single vitrified blastocyst transfer

Before the vitrified blastocyst transfer, the blastocyst quality was graded according to a previous report.^18^ The inner cell mass and trophectoderm were scored and categorized as good (AA, AB, BA), fair (BB, AC, CA), and poor (BC, CB, CC). The hatching status of the blastocyst at the time of transfer was also recorded as the following criteria: completely hatched, partly hatched, and not hatched. Single embryo transfer (SET) was performed in all cases.

### 2.6 Follow‐up and evaluation index

Implantation was confirmed when a gestational sac was visualized via an ultrasound examination, and clinical pregnancy was confirmed by the detection of a fetal heartbeat. LBR was calculated by dividing the live birth delivery cycles by the transfer cycles. The evaluated neonatal outcomes included the delivery method, weeks of gestational age, sex, birthweight, and neonatal malformations.

### 2.7 Study outcomes

The primary outcome was LBR. The secondary outcomes were the rate of complete hatching, CPR, and neonatal outcomes, including average gestational weeks, birthweight, malformation rate, and twin birth rate.

### 2.8 Statistical analysis

All statistical analyses were conducted using EZR (Saitama Medical Center, Jichi Medical University).^19^ Continuous variables are represented as means ± SD. The data were evaluated using chi‐square test, chi‐square test for contingency tables, and Mann‐Whitney U test to compare the LAH treatment group and the control. *P* < .05 was considered statistically significant. The patient characteristics including the woman's age at oocyte pickup and FET, previous failed cycles, a type of infertility, insemination methods, type of endometrial preparation and infertile cause were analyzed using propensity score matching conducted on adjusting for potential confounders in this study. It was judged to be balanced if the absolute value of the standardized mean difference (SMD) is <0.1.

### 2.9 Sample size estimation

It is difficult to define the sample size based on the published studies because there were no statistically significant differences in the effect of LAH on LBR using vitrified blastocysts.^8^ One study found a 13% difference in the LBR between the LAH treatment group and the control group.^20^ Based on that report, 558 transfer cycles (279 per group) are required to obtain an 90% chance of detecting (at a 5% significance level) an increase in the primary outcome measure from 28% in the control group to 41% in the LAH treatment group.

## 3 RESULTS

### 3.1 Patient characteristics

A total of 1180 FET cycles were performed during the study period. Of these, 482 cycles were excluded because of the exclusion criteria shown in Figure 1. The remaining 698 cycles, including 340 cycles in the LAH group and 358 cycles in control, were available for analysis and slight differences in women's age at FET (*P* = .14) and infertility cause (*P* = .08). All covariates were in good balance after propensity score matching. The 289 matched pairs were analyzed for differences in patient characteristics: 201 women who underwent the LAH treatment and 196 women in the control group; 42 women out of all experienced both treatments in different FET cycles (Table 1).

**TABLE 1:** Clinical characteristics among unmatched and propensity score–matched women

	Unmatched			Matched			
	LAH	Control	*P* Value	LAH	Control	*P* Value	SMD
FET cycle	358	340	–	289	289	–	–
Patient	230	216	–	201	196	–	–
Women's age at OPU	33.4 ± 4.0	33.7 ± 3.9	.42	33.7 ± 3.9	33.6 ± 4.0	.77	0.03
Women's age at FET	34.1 ± 4.1	34.6 + 3.9	.14	34.5 ± 4.0	34.4 ± 3.9	.77	0.03
Previous failed cycle	1.7 ± 1.9	1.8 ± 2.3	.37	1.8 ± 2.0	1.7 ± 2.1	.70	0.03
Type of infertility							
Primary	242 (67.6%)	215 (63.2%)	.26	186 (64.4%)	191 (66.1%)	.73	0.04
Secondary	116 (32.4%)	125 (36.8%)		103 (35.6%)	98 (33.9%)		
Insemination method							
cIVF	221 (61.7%)	210 (61.8%)	1.00	187 (64.7%)	181 (62.6%)	.67	0.04
ICSI	137 (38.3%)	130 (38.2%)		102 (35.3%)	108 (37.4%)		
Type of endometrial preparation							
Natural	268 (74.9%)	243 (71.5%)	.36	217 (75.1%)	210 (72.7%)	.57	0.06
HRT	90 (25.1%)	97 (28.5%)		72 (24.9%)	79 (27.3%)		
Infertile cause							
Oviduct factor	32 (8.9%)	42 (12.4%)	.08	28 (9.7%)	26 (9.0%)	.94	0.07
Endometrial factor	41 (11.5%)	32 (9.4%)		26 (9.0%)	29 (10.0%)		
Male factor	86 (24.0%)	68 (20.0%)		60 (20.8%)	64 (22.1%)		
Combination	8 (2.2%)	18 (5.3%)		7 (2.4%)	9 (3.1%)		
Unexplained	191 (53.4%)	180 (52.9%)		168 (58.1%)	161 (55.7%)		

Note

Values as mean ± SD or number (%).

SMD < 0.1 suggests adequate variable balance after propensity matching.

Abbreviations: FET, frozen embryo transfer; HRT, hormonal replacement therapy; LAH, laser‐assisted hatching; OPU, oocyte pickup; SMD, standardized mean difference.

### 3.2 Comparison of clinical outcomes

The clinical outcomes for the two groups are presented in Table 2. The rate of complete hatching after overnight culture was significantly higher in the LAH treatment group than in the control group (83.4% versus 2.1%, *P* < .01). The quality of the transferred blastocysts was comparable between the groups; however, significantly higher rates of implantation (46.0% versus 35.6%, *P* < .01), clinical pregnancy (40.8% versus 29.4%, *P* < .01), and live birth (34.3% versus 22.5%, *P* < .01) were observed in the LAH treatment group. A total of 99 babies were born in the LAH treatment group and 65 babies in the control group. All births were singleton live births, and no twinning was found in either group.

**TABLE 2:** Comparison of clinical parameters between the laser‐assisted hatching group and control group

	LAH	Control	*P* Value
Blastocyst warmed	290	290	–
Blastocyst survived	289 (99.7%)	289 (99.7%)	1.00
Blastocyst transferred	1	1	–
Culture after warming (hour)	18.3 ± 1.7	18.3 ± 1.6	.96
Transferred blastocyst status			
Completely hatched	241 (83.4%)	6 (2.1%)	<.01
Partly Hatched	36 (12.5%)	35 (12.1%)	
Not hatched	12 (4.2%)	248 (85.8%)	
Transferred blastocyst quality			
Good (AA, AB, BA)	236 (81.7%)	237 (82.0%)	.47
Fair (BB, AC, CA)	31 (10.7%)	26 (9.0%)	
Poor (BC, CB, CC)	22 (7.6%)	26 (9.0%)	
Implantation	133 (46.0%)	103 (35.6%)	.01
Clinical pregnancy	118 (40.8%)	85 (29.4%)	<.01
Live birth	99 (34.3%)	65 (22.5%)	<.01
Singleton live birth	99 (100%)	65 (100%)	1.00

Note

Values as mean ± SD or number (%).

*P* < .05 was considered statistically significant.

Abbreviations: FET, frozen embryo transfer; LAH, laser‐assisted hatching.

### 3.3 Comparison of neonatal outcomes

The neonatal outcomes of 153 children born after transfer are presented in Table 3. Live birth data were collected from 93 babies in the LAH treatment group and 60 babies in the control group. There were no differences between the LAH treatment and control groups in terms of delivery style, average gestational age weeks, the sex of the baby, or birthweight. One baby with muscular dystrophy was born in the LAH and a baby with a cleft lip in control. The rate of malformations did not differ between the groups.

**TABLE 3:** Comparison of perinatal outcomes between laser‐assisted hatching group and control group

	LAH	Control	*P* Value
Data collected from singleton live birth	93	60	–
Delivery style			
Vaginal delivered	68 (73.1%)	40 (66.7%)	0.47
Cesarean sections	25 (26.9%)	20 (33.3%)	
Gestational age week			
37‐41 wk	84 (90.3%)	50 (83.3%)	0.31
32‐36 wk	7 (7.5%)	9 (15.0%)	
≦31 wk	2 (2.2%)	1 (1.7%)	
Sex			
Male	49 (52.7%)	31 (51.7%)	1.00
Female	44 (47.3%)	29 (48.3%)	
Birthweight			
<1500 g	1 (1.1%)	1 (1.7%)	0.41
1500‐2499 g	10 (10.8%)	3 (5.0%)	
2500‐3999 g	82 (88.2%)	56 (93.3%)	
Malformation	1* (1.1%)	1** (1.7%)	1.00

Note

Values as mean ± SD or number (%).

*P* < .05 was considered statistically significant.

*muscular dystrophy and **cleft lip.

Abbreviation: LAH, laser‐assisted hatching.

## 4 DISCUSSION

The main question of the current study is whether the use of LAH in vitrified blastocyst improves the LBR and other associated clinical outcomes in the SET cycles. The findings show that the LAH procedure that consists of slicing a small section of the ZP contributed to the improvement in the LBR compared with the control group (*P* < .01). No multiple pregnancies were observed in either group. Our data show that LAH treatment for vitrified blastocyst in SET cycles has no adverse effects on obstetric or neonatal outcomes.

The ZP is a complex acellular matrix that surrounds and imparts essential developmental functions to both oocytes and early embryos. The ZP thickness in a matured oocyte is approximately 15 µm, and it acts as a gatekeeper of the sperm‐oocyte interaction during the fertilization event and as an embryonic protective barrier.^9^ The ZP of the blastocyst stage is thinned and lysed so that the embryo can escape from the ZP. This phenomenon is the most critical and essential event in implantation. IVF treatments, such as in vitro culture and freeze/thaw techniques, cause ZP hardening and reduce ZP proteolysis, therefore making it more difficult for the embryo to escape from the ZP.^21^, ^22^ Although many laboratories from the late 1980s to the early 2000s attempted chemical and mechanical techniques for AH to resolve these issues, the 1.48 µm, infrared diode laser system has been widely used nowadays. The easy laser procedure can open the small hole in the ZP safely, and many operators prefer to use the LAH method for the recent ART technologies.

The chromosome abnormalities in embryos increase with age. Greater 50% of embryos have an extra or missing chromosome in women 40 years and older, and most of them will be miscarried even if they get pregnant. Therefore, it is a limited effect in advanced age women even if their vitrified blastocysts are treated with LAH. We show the great benefit of the LAH by slicing off a small section of the ZP, excluding the potential risk of maternal age in this study.

Many published reports have suggested that AH treatment does not improve clinical outcomes; however, they excluded a comparison of different AH techniques and protocols, the different stages of the embryo, the cycle type, or the indications for the procedure.^8^ However, in studies focusing on LAH, a surprising benefit was found when LAH was used along with a vitrified blastocyst, in which the ZP was widely opened (around a one‐quarter to one‐half opening of the ZP) with multiple laser shots,^20^, ^24^ or was removed entirely.^21^ Alternatively, LAH was not effective on ZP hole opening, drilling, or thinning when the laser was applied on cleavage‐stage embryos or blastocysts in the FET cycle.^8^ Also, no benefits were found for L AH with a ZP opening of any size in fresh embryos, including those in the blastocyst stage.^8^

In this study, LAH was performed during the warming procedure in a dilution solution. The procedure was completed within 2 minutes, with still enough time to finish the work during blastocyst warming. Blastocyst warming in a dilution solution resulted in a large perivitelline space, which allowed a small section of the ZP to be sliced easily and safely using a noncontact laser beam. Hiraoka et al^23^ were the first to report that ZP removal using multiple laser shots and mechanical pipetting improved the clinical outcomes of vitrified blastocysts compared with a single‐hole ZP opening and the control group. After LAH treatment, the ZP‐free blastocyst increases the expression of integrin, which is the fibronectin receptor and is considered the critical outgrowth for blastocyst attachment and adhesion to the endometrial epithelium.^25^ The current study suggests that 83.4% of LAH blastocysts completely escaped from the ZP after overnight culture and improved the CPR and LBR compared with the control group. Extending the culture of vitrified blastocysts would be helpful for measuring embryonic development and improving CPR and LBR.^26^ Also, these did not affect either the clinical or neonatal outcomes in the current study.

After escaping the ZP, the blastocyst is sticky, which may provide a definite advantage in terms of increasing the chance of cell attachment to the endometrium of the uterus after transfer. Simultaneously, this stickiness is considered a significant disadvantage in the laboratory, where it tends to adhere tenaciously to the surface of the plastic culture dish, handling pipette, and embryo transfer catheter.^9^ Therefore, some embryologists prefer not to handle hatched blastocysts and avoid the potential risks involved with handling one. To resolve this issue, the use of inorganic or organic macromolecules, such as polyvinyl alcohol or albumin, is useful. In this study, all naked blastocysts were handled using PVP‐coated pipette and enriched albumin medium; therefore, we did not have an issue with adhesion to the equipment. We consider reliable embryo handling to be an essential key for a successful transfer, which can result in high CPR and LBR.

The follow‐up studies have been conducted to evaluate the effect of AH, including fresh cycles of cleavage‐stage embryos treated with acidic Tyrode's solution^27^, ^28^ and laser for ZP drilling,^29^ and frozen cycles on cleavage‐stage embryos treated with laser ZP thinning^30^, ^31^ and on blastocyst stage treated with laser ZP thinning or bleaching.^32^ AH would be considered a safe method of ART because it has no adverse effects on obstetrical and neonatal outcomes, including congenital malformation.^30^, ^32^, ^33^ We evaluated neonatal outcome data of vitrified blastocysts with LAH from 93 babies and from 60 babies in the control group. No differences in the average gestational weeks, sex of the child, birthweight, or malformations of the newborn were found that are consistent with a previous report.^34^ In addition, no monozygotic twins occurred in the current study, which used a single blastocyst transfer in FET cycles. The risk of monozygotic twinning would not be increased in widely ZP opening but in ZP drilling and thinning.^33^, ^35^, ^36^ However, the overall number of reports is small.

This study was analyzed the propensity score–matched data, and the potential biases were removed. However, difficulty in removing the bias could also be a limitation. The blastocyst diameter before vitrification might be a possible factor to affect the clinical outcomes. However, the proportion of blastocysts with a diameter greater than 150 µm before vitrification was the same (73.0% in both groups); thus, it would have little effect on the results in this study. When introducing the LAH as a routine procedure, we had doubted the effectiveness of AH because ASRM has reported that it has a limited effect on IVF patients. In this study, we evaluated the effectiveness of LAH treatment retrospectively, and patients with or without LAH treatment for the vitrified blastocyst were compared. In terms of blastocyst quality, it was similar between the LAH treatment group and control group, including 81.7% with the LAH group and 82.0% with the control group of vitrified blastocysts, which were graded “Good” after overnight culture (Table 2). It would be the great advantage of this study, and our practices of vitrification/warming and LAH did not affect embryonic development. Kirienko et al^37^ evaluated the quality of post‐warmed blastocysts and concluded that the mechanical removal of ZP did not affect the clinical outcomes after vitrified‐warmed blastocyst transfer. However, it seems to have the potential bias of embryo quality because the good quality rate of transferred blastocyst was approximately 40% or less.

One may have a concern about comparing the clinical outcomes between the LAH and control groups, because the LAH group was from July 2016 to May 2018 while the control group from June 2014 to September 2016. Considering the laboratory bias, our laboratory had been operating without any change during the experimental period. All embryos were cultured in a one‐step medium using the benchtop CO_2_ incubators and vitrified using the open‐system Cryotop. Fully trained embryologists with over 10 years of experience conducted all procedures, and a physician is a specialist in the field of reproductive endocrinology and infertility performed all embryo retrievals and transfers. However, we cannot completely ignore the influence of the different observation periods on the clinical outcomes.

In conclusion, this study was evaluated the effect on clinical and neonatal outcomes of the use of multiple laser beams to slice a small section of the ZP in patients undergoing SET of vitrified blastocysts using propensity score–matched data. The results show that LAH has clear benefits for clinical outcomes. The integration of the method of SET in the vitrified blastocyst transfer cycle and slicing off a small section of the ZP using multiple laser beams may actively improve the IVF success rate. Further studies are needed whether LAH to the vitrified blastocysts improves the CPR and LBR.

## DISCLOSURES

*Conflict of Interest*: Yuji Endo, Shingo Mitsuhata, Momoko Hayashi, Yoshitaka Fujii, and Hiroaki Motoyama declare that they have no conflict of interest. *Human Rights Statement and Information*: All procedures followed were in accordance with the ethical standards of the responsible committee on human experimentation (institutional and national) and with the Helsinki Declaration of 1964 and its later amendments. Informed consent was obtained from all patients for being included in the study. *Animal Studies*: This article does not contain any studies with animal subjects performed by any of the authors.

## ETHICAL APPROVAL

The study design was approved by the appropriate ethics committee of Kurashiki Medical Clinic, Okayama, Japan.

## CLINICAL TRIAL REGISTRY

Not applicant.
